# The mitochondrial genome of *Suillia* sp. (Diptera: Heleomyzidae)

**DOI:** 10.1080/23802359.2019.1644222

**Published:** 2019-07-22

**Authors:** Dongyue He, Yue Liu, Liang Wang, Xin Li, Ding Yang, Chen Lin

**Affiliations:** a College of Plant Protection, China Agricultural University, Beijing, China;; b Institute of Life Science and Technology, Inner Mongolia Normal University, Huhhot, Inner Mongolia, China

**Keywords:** Mitochondrial genome, Heleomyzidae, *Suillia*, phylogeny

## Abstract

The mitogenome of *Suillia* sp. was sequenced as the first representative of the family Heleomyzidae. The mitogenome is 15,660 bp totally, consisting of 13 protein-coding genes, 2 rRNAs, and 22 transfer RNAs. The nucleotide composition biases toward A and T is 75.1％ of the entirety. All PCGs start with ATN codons except COI and ND1 and end with TAA or incomplete stop codon. Phylogenetic analyses based on 9 dipteran species supported the relationship of Opomyzoidea + (Ephydroidea + (Lauxanioidea + (Sciomyzoidea + (Sphaeroceroidea + Tephritoidea)))).

## Introduction

Heleomyzidae is a small family with body size from minute to large (2.0–14.0 mm). Adults of this family are often distinctly pruinose and yellow, reddish yellow, or reddish brown to black in color (Gordon and Peterson 1987). The heleomyzid flies, which have about 800 described species worldwide, are placed in superfamily Sphaeroceroidea (Pape et al. 2011). Some species of *Suillia* are recorded as pests of onion and garlic (Papp 1998).

The adult specimens of *Suillia* sp. (accession number: YDHELE) used for this study were collected from Wulingshan National Nature Reserve (40.3318°N, 117.2922°E, 975 m), Xinglong, Hebei, China. The specimens were identified by Ding Yang and deposited in the Entomological Museum of China Agricultural University (CAU).

The genomic DNA was extracted from adult’s whole body using the DNeasy DNA Extraction kit (TIANGEN) and stored at −20 °C refrigerator. The library was sequenced on an Illumina HiSeq 2500. The bait sequence COI was amplified by standard PCR reactions and BLAST search was carried out with BioEdit 7.0.5.3. and the position of all tRNA genes was confirmed using tRNAscanSE 2.0 (Lowe and Chan 2016). The nearly complete mitogenome of *Suillia* sp. (MN026917) is 15,660 bp in length and consists of 13 typical invertebrate PCGs, 22 transfer RNA genes, 2 rRNA genes (12S and 16S), and part control region, which were similar to other Diptera flies reported before (Li et al. 2016; Zhou et al. 2017; Qilemoge Gao et al. 2018; Ren et al. 2019). The nucleotide composition of the mitogenome was biased toward A and T, with 75.1% of A + T content (A = 38.8%, T = 36.3%, C = 14.9%, G = 9.9%). Among the protein-coding genes, five genes took the start codon of ATG, five genes used ATT and one gene (ND2) used ATA as a start codon, while COI gene and ND1 gene got CCG and TTG, respectively. The termination codon of these protein-coding genes had four types (eight genes used TAA, CYTB gene used TAG, ND2 gene used incomplete stop codon TA + tRNA, two genes used T + tRNA and ND1 missed normal termination codon).

**Figure 1.**
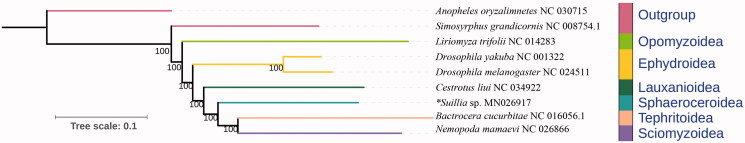
The phylogenetic tree of ML analysis based on 13PCGs; “*” indicated new sequenced data in this study.

There are 8 species retrieved from NCBI and 1 new sequenced datum used in phylogenetic analysis. The genbank accession numbers are listed as follows: *Anopheles oryzalimnetes* NC_030715, *Bactrocera cucurbitae* NC_016056.1, *Ceratitis capitata* NC_000857, *Drosophila melanogaster* NC_024511, *Drosophila yakuba* NC_001322, *Liriomyza trifolii* NC_014283, *Nemopoda mamaevi* NC_026866, *Simosyrphus grandicornis* NC 008754.1, *Suillia sp. MN026917. Thirteen protein-coding genes (PCGs) were used to reconstruct the phylogenetic relationship with maximum likelihood method. The topology was given and bootstrap support numbers were shown in Figure 1. ML analysis revealed that Sphaeroceroidea was assigned to be the sister of the clade of Tephritoidea + Sciomyzoidea. The higher-level relationship of Opomyzoidea + (Ephydroidea + (Lauxanioidea + (Sciomyzoidea + (Sphaeroceroidea + Tephritoidea)))) was supported.

The complete mitochondrial genome of *Suillia* sp. provides valuable information for future genetic and evolutionary studies of family Heleomyzidae and superfamily Sphaeroceroidea.
